# Elevated secondary infection rates in patients with coronavirus disease 2019 (COVID-19) requiring extracorporeal membrane oxygenation

**DOI:** 10.1017/ice.2021.61

**Published:** 2021-02-09

**Authors:** Joseph E. Marcus, Valerie G. Sams, Alice E. Barsoumian

**Affiliations:** 1 Infectious Disease Service, Department of Internal Medicine, Joint Base San Antonio Fort Sam Houston, San Antonio, Texas; 2 Department of Surgery, Brooke Army Medical Center, Joint Base San Antonio Fort Sam Houston, San Antonio Texas

Coronavirus disease 2019 (COVID-19) has had a tremendous impact in healthcare, including a surge of hospitalizations. To prevent in-hospital outbreaks, contact and airborne precautions have been implemented for patients with COVID-19 with demonstrated success in preventing severe acute respiratory coronavirus virus 2 (SARS-CoV-2) transmission.^1^ Data on the rates of secondary infections in patients hospitalized with COVID-19 are limited.^2^ Although early studies showed low rates of secondary infections in patients with COVID-19,^3^ more recent studies in both larger and sicker populations have shown elevated rates of secondary infection.^4–6^

Extracorporeal membrane oxygenation (ECMO) is used for pulmonary bypass in patients with reversible respiratory failure, and it poses a significant risk of secondary infections.^7^ ECMO is currently only recommended for COVID-19 patients with few comorbidities and without severe multisystem organ failure.^8^ Although it has been utilized worldwide for patients with COVID-19, no data on secondary infections in these patients are available.^9^ In this study, we retrospectively compared secondary infection rates on ECMO for patients with COVID-19 to patients with influenza. Although both viruses can cause devastating pulmonary disease, unlike influenza, patients hospitalized with COVID-19 are treated with immunosuppression. Additionally, COVID-19 has been associated with high patient volumes, which strained healthcare systems. As such, we hypothesized that there would be more secondary infections with COVID-19, despite the increased use of PPE and emphasis on infection prevention.

## Methods

All patients who completed a course of ECMO at Brooke Army Medical Center between January 1, 2013, and October 10, 2020, with confirmed influenza or severe acute respiratory coronavirus virus 2 (SARS-CoV-2) were included in this retrospective analysis. Positive cultures during ECMO course or within 48 hours of decannulation that were determined to be pathogenic by the patient’s treatment team were labeled as bloodstream, respiratory, or urinary infections based on the site of culture. Culture organisms that were considered colonizers or contaminants by the treatment team were excluded. Multidrug-resistant organisms (MDROs) were defined as resistance to 3 or more classes of antibiotics.

We compared patients with influenza and COVID-19 by demographics, duration of hospitalization prior to ECMO cannulation, length of stay, mortality, number of infections, infection rates per 1,000 ECMO patient days, and MDRO rate. Nominal variables and rates were compared using the χ^2^or Fisher exact test as appropriate, whereas continuous variables were compared by Mann-Whitney *U* test. A *P* value of .05 was considered significant.

## Results

Of the 210 patients who received ECMO during the study period, 39 patients (19%) were diagnosed with either COVID-19 or influenza. All patients received the venovenous modality of ECMO. Overall, 4 patients (10%) who completed their ECMO course were still inpatients and 35 patients (90%) had completed their hospital course as of October 10, 2020, with a survival rate to hospital discharge of 72%.

We detected minimal differences in the demographics of patients who underwent ECMO with influenza versus COVID-19 (Table 1). All patients with COVID-19 were treated with immunosuppression during their hospital course. Patients with COVID-19 were hospitalized longer prior to ECMO cannulation than patients with influenza (median 12 [IQR, 8–14] days vs 5 [IQR, 3–8]; *P* = .001).

**Table 1.** Characteristics of Patients Receiving Extracorporeal Membrane Oxygenation (ECMO) With Influenza or COVID-19

Characteristic	All	Influenza (n=22)	COVID-19 (n=17)	*P* Value^a^
Age, median y (IQR)	43 (34–53)	45 (32–55)	42 (35–49)	.76
Sex, male, no. (%)	28 (70)	15 (68)	13 (76)	.72
**Comorbidities, no. (%)**				
Obesity	25 (63)	15 (68)	10 (58)	.51
Hypertension	13 (33)	8 (36)	5 (29)	.74
Diabetes mellitus	11 (28)	6 (27)	5 (29)	1
Days in hospital before ECMO, median d (IQR)	8 (4–13)	5 (3–8)	12 (8–14)	.001
Hours on ECMO, median h (IQR)	360 (200–610)	360 (196–604)	321 (191–441)	.93
Days of hospitalization, median d (IQR)	38 (27–47)^b^	36 (28–43)	38 (28–54)^b^	.43
Survival to discharge, no. (%)	26 (67)^b^	17 (89)	9 (69)^b^	.46
Days to first ECMO infection after cannulation, median d (IQR)	8 (5–16)	18 (10–21)	5 (3–7)	.01
Days to first ECMO infection after hospitalization, median d (IQR)	20 (14–26)	21 (16–25)	19 (14–26)	.92
**Patients with infection, no. (%)**				
Any infection	18 (46)	8 (36)	10 (58)	.22
BSI	13 (33)	6 (27)	7 (41)	.60
RI	7 (18)	2 (9)	5 (29)	.21
UTI	1 (3)	1 (5)	0	
**Infections per 1,000 ECMO days**				
Total	27.2	17.7	37.3	.04
BSI	16.8	13.2	21.8	.31
RI	9.1	4.4	15.6	.19
UTI	1.3	2.2	0	.79
Multidrug-resistant bacteria	5/17 (29)	1/6 (17)	4/11 (36)	.6

Note. IQR, interquartile range; BSI, bloodstream infection; RI, respiratory infection, UTI, urinary tract infection.

a Presented as no. (%) or median (IQR).

b χ^2^, Fisher exact, or Wilcoxon rank-sum test.

c Excludes 4 patients with COVID-19 who were still inpatients as of October 10, 2020.

For the primary outcome, patients with COVID-19 had greater rates of secondary infection while on ECMO (37.3 per 1,000 patient days vs 17.7; *P* = .04). Infections occurred earlier after cannulation in patients with COVID-19 (median day 5 [IQR, 3–8] vs 16 [IQR, 10–21]; *P* = .03). However, there was no difference in day of infection after hospital admission (19 [IQR, 14–26] vs 21 [IQR, 16–25]; *P* = .92). MDROs were isolated at similar frequencies in the 2 groups (17% vs 36%; *P* = .60).

## Discussion

In this study, we compared patients with respiratory viruses requiring ECMO, and we detected an elevated secondary infection rate for patients with COVID-19. The reasons for this difference are likely multifactorial and include strain on the healthcare system, the use of immunosuppressants, and possible COVID-19 disease-specific characteristics. Overall, the rate of infections of 37.1 per 1,000 patient days in patients with COVID-19 is higher than the national average for all adults who receive ECMO of 30.6.^7^

Infections tended to occur earlier in the ECMO course for patients with COVID-19 than for patients with influenza. Despite similar time on ECMO circuit between the 2 groups, no patient with COVID-19 had an infection after ECMO day 17, whereas 5 infections occurred after that day in patients with influenza. Additionally, patients with COVID-19 had longer hospital courses pre-ECMO, which may contribute to the timing of infection onset. Further studies are needed to evaluate the risk factors contributing to secondary infection.

This study has several limitations. It was a retrospective, single-center study with a small number of patients, and it may be underpowered to detect differences in specific types of infections and MDROs. Secondly, COVID-19 and influenza have different pathophysiology and comparisons may be premature. We were not able to differentiate whether secondary infections are caused by a failure of infection prevention practices, overall strain on the healthcare system, or due to differences in the underlying disease process. Finally, we have no data on adherence to PPE and hand hygiene.

In this study, we compared critically ill patients that presented with similar demographics to an established ECMO center with adequate resources throughout the pandemic. Our results show that the risk of secondary infections is significant for this population. Infection control strategies should continue to be implemented that protect healthcare workers, with emphasis on adherence to infection prevention and control bundles. However, secondary infections for COVID-19 patients on ECMO may persist due to unrecognized factors. Larger, multicenter trials with COVID-19 patients are needed to determine the best practices for caring for these patients to reduce secondary infections.
